# Effects of an interdisciplinary programme on psychoemotional factors in informal caregivers of people with Alzheimer’s disease

**DOI:** 10.3389/fpsyg.2025.1524292

**Published:** 2025-03-07

**Authors:** Laura Muñoz-Bermejo, Javier Urbano-Mairena, Violeta Calle-Guisado, Cristina Mendoza-Holgado, María del Rocío Jerez-Barroso, Belén Suárez-Lantarón, José Francisco López-Gil, Sabina Barrios-Fernández

**Affiliations:** ^1^Social Impact and Innovation in Health (InHEALTH) Research Group, University Centre of Mérida, University of Extremadura, Mérida, Spain; ^2^Department of Anatomy, Cell Biology and Zoology, Faculty of Medicine and Health Sciences, University of Extremadura, Badajoz, Spain; ^3^Education Sciences Department, Faculty of Education, University of Extremadura, Badajoz, Spain; ^4^One Health Research Group, Universidad de Las Américas, Quito, Ecuador

**Keywords:** informal caregivers, care overload, quality of life, occupational balance, AD, familiar functioning, intervention

## Abstract

Alzheimer’s disease (AD) causes a progressive deterioration in the person’s memory and cognitive function, leading to a greater degree of dependency as the disease progresses. This causes a progressive increase in caregiver overload, leading to physical, psychological, and social impairments. This study aimed to evaluate the effects of a nine-month interdisciplinary intervention covering three areas (cognitive-behaviour, psycho-emotional, and physical activity) on various psycho-emotional variables in informal caregivers of people with AD. A trial was conducted in which 59 informal caregivers of people with AD were administered various instruments to assess psychoemotional aspects (EuroQol-5 Dimensions-3 Levels, Zarit Burden Inventory test, Satisfaction with Life Scale, General Happiness Questionnaire, Occupational Balance Questionnaire, Rosenberg Self-esteem Scale, Duke-UNC-11 Functional Social Support Questionnaire, and the Family Apgar Scale). Significant between-group improvements were obtained in occupational balance at 3 (*p* = 0.002), 6 (*p* = 0.013) and 9 months (*p* = 0.022) of intervention, in perceived social support at 3 months (*p* = 0.043) and satisfaction with life at 6 months (*p* = 0.020). No significant between-group differences were found in the remaining variables, although there were positive trends in intra-group scores for caregiver overload, quality of life, life satisfaction and family functioning. Thus, an interdisciplinary programme could have a positive influence on the variables assessed in informal caregivers of AD.

## 1 Introduction

Alzheimer’s disease (AD) cases are expected to reach 152.8 million by 2050 (Collaborators GBDDF, 2022). This neurodegenerative disease causes a progressive deterioration in the person’s memory and cognitive function, and there is currently no curative treatment (Williams et al., 2021). Therefore, early detection and diagnosis and individualised, evidence-based treatment are essential to minimise the disease course and symptomatology for the individual. However, during the AD progression, the individuals become increasingly dependent, increasing the burden on the caregiver significantly, which can have physical, psychological and social effects (Atri, 2019).

The burden of care often lies with family members, experiencing challenges associated with social isolation, poor physical health, psychological disorders such as depression and financial difficulties (Brodaty and Donkin, 2009). An informal caregiver is a person who provides some kind of continuous care, usually unpaid, to a person with a chronic disease. This assistance is primarily related to Activities of Daily Living (ADLs), which include bathing, toileting and hygiene, feeding, dressing and mobility; and to Instrumental Activities of Daily Living (IADLs), which include activities related to home care and community use, such as financial assistance, transportation, shopping, cooking, cleaning, and medication administration, among others (Allen et al., 2019). Approximately two-thirds of caregivers of people with AD are women (Freedman and Spillman, 2014; Rabarison et al., 2018) and usually live with the person with the disease (Kasper et al., 2015). Therefore, the burden of caregiving has a direct impact on the quality of life of these women. Quality of life refers to the physical and psychological health, social and economic aspects, personal goals and expectations that have an impact on people’s daily lives (Martens and Addington, 2001). Caregivers must cope with disruptive behaviours of the dependent person, mood disorders, family conflicts, and the provision of basic care, among others, which imply an increased risk of physical and psychological distress, including cardiovascular diseases, depression and anxiety due to stress and burnout (Cheng, 2017). Moreover, depression is more prevalent in caregivers of people with dementia than in other caregivers who provide care to people with schizophrenia or stroke (Sallim et al., 2015; Thunyadee et al., 2015).

The burden of care can affect the caregiver’s occupational balance, defined as the positive balance between different meaningful activities, i.e., those that a person does or expects to do as work or leisure activities (Park et al., 2021; Lee et al., 2023; Evans, 1987). Furthermore, 57% of employed people caring for a person with AD reported that they needed to leave early or arrived later compared to carers with other conditions (Association As, 2024). 18% of caregivers reduced their working hours due to the responsibility of caring for the person with AD, which impacts their professional development and therefore their perceived self-esteem, understood as the positive or negative feelings and perceptions associated with the performance of different life functions (Bhattacharjee et al., 2012). In this sense, stressors can affect subjective perceptions of happiness and life satisfaction, two vital components of caregivers’ well-being (Lin et al., 2010).

Happiness can be considered as a mental state or feeling characterised by pleasure or satisfaction. For years, the World Health Organisation has been making efforts to address this issue as a component of health (Lyubomirsky et al., 2005). The concept of happiness is also related to life satisfaction or evaluation, subjective well-being, psychological well-being, quality of life or affect (Cho, 2017). Previous studies (An et al., 2020; Kim et al., 2022; Milovanska-Farrington and Farrington, 2022) have measured happiness, life satisfaction or well-being in the general population, but few articles have focused on special groups, such as people with disabilities and their caregivers in society.

Social aspects should also be considered when assessing the quality of life of the informal caregiver. There are two types of social support, received social support is the objective quantification of the help received by the social environment; perceived social support is measured in terms of the extent and quality of support perceived by the caregiver from their environment, and directly impacts on psychological burden relief (Ong et al., 2018; Nemcikova et al., 2023). Thus, greater emotional support from family and friends of the caregiver may imply greater caregiving satisfaction, so caregiver-perceived social support and family bonding may positively influence caregiving (Leggett et al., 2021).

Different types of caregiver intervention programmes are found in the literature, including psychoeducational (de Rotrou et al., 2011; Morhardt et al., 2019; Meziane-Damnée et al., 2023), psycho-emotional (Cheng et al., 2019; Wiegelmann et al., 2021), cognitive-behaviour (Gomez Gallego and Gomez, 2017; Kim, 2020), and interventions that include physical exercise (De la Rosa et al., 2020; Cui et al., 2018). Thus, the need to support the quality of life and well-being of informal caregivers is highlighted, as well as the importance of analysing the impact of these programmes and the support they provide for caregiving. For all the above reasons, this study aims to assess the effects of an interdisciplinary intervention (based on cognitive-behaviour, psycho-emotional, and physical activity) on psychoemotional variables such as overload, quality of life, happiness, life satisfaction and occupational balance in informal caregivers of people with AD. The ultimate goal is to provide psycho-educational skills and resources to help and improve their emotional and psychological state, thereby increasing the caregiver’s quality of life and satisfaction.

## 2 Materials and methods

### 2.1 Study design

A clinical trial was conducted to assess the effects of a nine-month interdisciplinary programme with informal caregivers of people with AD. This study is part of the Cost-Effectiveness Programme, designed to evaluate the cost-effectiveness of an interdisciplinary programme for informal caregivers of people with AD. The protocol of this study was previously published (Muñoz-Bermejo et al., 2022).

### 2.2 Participants

The sample was composed of a total of 59 informal caregivers of people with AD belonging to associations of family members of individuals with dementia, with 12 of the participants being men and 47 women. Two initial groups were established by convenience, an intervention group (IG) of 32 people (26 women and 6 men) and a control group (CG) of 27 participants (21 women and 6 men). The mean age of the IG participants was 59.2 ± 10.2 years, while that of the CG was 59.5 ± 11.2 years.

The following eligibility criteria were established: being an informal primary caregiver of a person with AD, by providing care more than 20 h per week, for more than 3 months, and with the will to continue for at least the next 12 months; not suffering from pathologies that contraindicate physical activity or special adaptations such as coronary pathologies, thrombosis, symptoms associated with COVID-19, among others; not having participated in any physical exercise programme in the 3 months before the intervention; not having received psycho-educational or cognitive-behaviour sessions in the 3 months before the intervention; having signed the informed consent form for the study and given it to a member of the research team.

### 2.3 Intervention

The IG participated in a 9-month intervention. During this time, participants attended two weekly face-to-face sessions. They also accessed virtual sessions where they could visualise the contents covered in a summarised way. Participants had access to these virtual sessions through the online platform created for Integral-Care.^1^ The subjects, in addition to being able to view brief sessions on the different areas of the intervention, had access to various documents with information related to the contents addressed during the face-to-face sessions.

These sessions were divided into three intervention areas: Cognitive-Behaviour area or Health Education, Psycho-emotional Area, and Physical Activity. A total of 60 face-to-face sessions were held (20 sessions per area) addressing a different area each week (Table 1, Distribution of programme contents).

**Table 1:** Distribution of programme contents.

Intervention areas	Contents
Cognitive—behavioural (Health education)	Alzheimer’s disease Patient care: mouth, skin, and digestive tract
Psycho-emotional	Caregiver satisfaction and happiness Occupational balance Caregiver’s emotions and roles
Physical activity	Postural hygiene and breathing Corporal mobility Functional exercise: upper and lower body strength-building

Conversely, the participants in the CG did not participate in any intervention.

### 2.4 Data collection

Participants in both the IG and CG groups took an assessment at baseline and 3, 6 and 9 months after the intervention (from October 2022 to June 2023), plus an additional one at 1 month after the end of the intervention (July 2023). Participants were contacted before the assessment, were given 1 week to complete the questionnaires, and were encouraged to complete them if they were not completed when the period expired.

### 2.5 Instruments

#### 2.5.1 Socio-demographic data

A socio-demographic questionnaire was used to collect information on the degree of relationship to the person with AD, age, and sex.

#### 2.5.2 Psychoemotional outcomes

##### 2.5.2.1 Quality of life

The EQ-5D-3L questionnaire assesses participants’ state of health. It is composed of three parts: (1) a descriptive system with three severity levels, assessing several health dimensions (mobility, self-care, activities of daily living, pain/discomfort and anxiety/depression) with a one (no problems) to three (external problems/impossibility) scale; (2) a visual analogue scale; and (3) a social values index generated from the health states obtained in the first level (Herdman et al., 2011). This instrument has demonstrated validity and reliability in both young (Milovanska-Farrington and Farrington, 2022) and adult populations (Ong et al., 2018).

##### 2.5.2.2 Caregiver overload

The Zarit Burden Inventory (ZBI; Spanish version) was used to assess caregiver strain. It is consists of 22 items in a Likert scale format, which quantify caregiver overload using a scale from one (never) to five (almost always). The sum of all items reflects the degree of caregiver overload (Zarit et al., 1980). The scale showed a Cronbach’s alpha of 0.84 in the Spanish population (Nemcikova et al., 2023; Leggett et al., 2021). The abbreviated Zarit scale demonstrated its validity and reliability among caregivers of individuals with various conditions (de Rotrou et al., 2011).

##### 2.5.2.3 Happiness and life satisfaction

The General Happiness Questionnaire measures the subjective happiness of the participants. It is composed of four items on a Likert scale (Lyumbornirsky and Lepper, 1999).

The Satisfaction with Life Scale (SWLS; Spanish Version) was used to measure overall life satisfaction, consisting of five items on a scale from one “strongly disagree” to five “strongly agree”(Diener et al., 1985). The reliability index calculated using Cronbach’s alpha suggested that the scale has very good internal consistency (Cronbach’s alpha = 0.84; De la Rosa et al., 2020). This instrument has proved to be a reliable and valid measure of overall life satisfaction in the general population (Muñoz-Bermejo et al., 2022; Herdman et al., 2011).

##### 2.5.2.4 Satisfaction with occupations and occupational balance

The Occupational Balance Questionnaire (OBQ-E) allows the assessment of participants’ satisfaction with their occupations, using 13 items, answered on a Likert scale from cero “strongly disagree” to five “strongly agree” (Gómez, 2017). The OBQ-E has shown a good internal consistency (Cronbach’s alpha = 0.87), being a valid a reliable measurement instrument in Spanish adults (Gómez, 2017).

##### 2.5.2.5 Self-esteem

Rosenberg Self-Esteem Scale (EAR) used for self-esteem measurement, through 10 items with content centred on feelings of self-respect and self-acceptance. This scale demonstrates high internal consistency (Cronbach’s alpha = 0.87). The Spanish version used has good validity and reliability data (Morejón et al., 2004).

##### 2.5.2.6 Perceived social support

The Duke-UNC Functional Social Support Questionnaire consists of 11 items and a Likert scale from one “much less than I would like” to five “as much as I would like” (Broadhead et al., 1988). An internal consistency of 0.90 was shown in the Spanish population, confirming the reliability of this instrument in this population (Broadhead et al., 1988).

##### 2.5.2.7 Family functioning

The Family Apgar scale allows the assessment of the perception of family functioning, with five items with a Likert scale from zero “almost never” to two “almost always” (Hernández-Gúzman et al., 2009). It has proven to be a reliable and appropriate instrument for assessing older people (Nikzad-Terhune et al., 2019; Hernández-Padilla et al., 2021), indicating an internal consistency of 0.84 (Cronbach’s alpha; Hernández-Gúzman et al., 2009).

### 2.6 Statistical analysis

Methods such as density and quantile-quantile plots were used to evaluate the normal distribution of the variables, supplemented by the Shapiro–Wilk test. Baseline characteristics of study participants are shown as means (standard deviation) for continuous variables and numbers (percentages) for categorical variables. Using a per-protocol analysis (i.e., including only those participants who complete the entire study), data were displayed as change from baseline (when intervention started) to 3, 6, 9 months and follow-up (10 months) for each group, determined by the mean difference and its 95% confidence interval (CI). The effects of the intervention on the primary endpoint (i.e., EQ-5D-3L score) and secondary endpoints (i.e., Zarit Burden Inventory test score, Satisfaction with Life Scale score), General Happiness Questionnaire score, Occupational Balance Questionnaire score, Rosenberg self-esteem scale score, Duke-UNC-11 Functional Social Support Questionnaire score, Family Apgar scale score were assessed. For continuous variables, between-group and within-group differences of both primary and secondary endpoints were determined by unpaired *t-*test or paired *t*-test, respectively. For categorical variables, between-group differences were determined by the chi-squared (*χ*^2^) test. All statistical analyses were performed using R statistical software (version 4.3.2) developed by the R Core Team in Vienna, Austria, and RStudio (2023.09.1 + 494) from Posit in Boston, MA, United States. A *p* value <0.05 was chosen for statistical significance.

## 3 Results

Table 2 shows the basic characteristics of the sample. No significant differences were found in any of the variables analysed. The main differences were found in Rosenberg’s Self-esteem Scale, without being significant (*p* = 0.062).

**Table 2:** Baseline characteristics of the participants.

Variables	Total, n (%)	Missing, n		IG	CG	*p*
Sociodemographic						
Sex	59 (98.3)	1	Men (%)	6 (18.8)	6 (22.2)	0.996
			Women (%)	26 (81.2)	21 (77.8)	
Age (years)	59 (98.3)	1	Mean (SD)	59.2 (10.2)	59.5 (11.2)	0.908
Primary endpoint						
EQ-5D-3L (score)	59 (98.3)	1	Mean (SD)	83.2 (21.1)	83.2 (16.1)	1.000
VAS (score)	59 (98.3)	1	Mean (SD)	68.2 (22.6)	74.0 (17.3)	0.283
Secondary endpoints						
Zarit Burden Inventory test (score)	59 (98.3)	1	Mean (SD)	19.3 (5.8)	18.8 (7.1)	0.768
Satisfaction with Life Scale (score)	39 (65.0)	21	Mean (SD)	17.8 (4.7)	18.3 (4.1)	0.678
General Happiness Questionnaire (score)	59 (98.3)	1	Mean (SD)	4.4 (1.0)	4.4 (0.9)	0.892
Occupational Balance Questionnaire (score)	50 (83.3)	10	Mean (SD)	48.4 (17.0)	54.0 (12.9)	0.166
Rosenberg self-esteem scale (score)	59 (98.3)	1	Mean (SD)	31.1 (5.4)	33.8 (4.4)	0.062
Duke-UNC-11 Functional Social Support Questionnaire (score)	59 (98.3)	1	Mean (SD)	39.4 (10.1)	39.3 (10.2)	0.967
Family Apgar scale (score)	59 (98.3)	1	Mean (SD)	8.0 (2.5)	7.2 (3.4)	0.313

BMI, body mass index; CG, control group; EQ-5D-3L, EuroQol-5 Dimensions-3 Levels; IG, intervention group; SD, standard deviation; VAS, visual analogue scale. ^†^According to the World Health Organisation criteria (WHO, 2000).

Table 3 indicate the EQ-5D-3L scores for both the three-level descriptive system and the visual analogue scale (VAS). Although no significant differences were found in any of the evaluation moments, an improvement in the IG scores with respect to the CG was observed in both parts of the questionnaire. In the descriptive system, IG scores (pre-intervention = 83.2) improve at 6 (80.5 vs. 83) and 9 months (80.9 vs. 86.2) with respect to CG (pre-intervention = 83.2). Although there were no significant differences, the IG scored better in all evaluations with respect to the initial score (68.2), while the CG scored worse in all evaluations with respect to the initial score.

**Table 3:** Results of study variables by group at 3-, 6-, 9-, and 10-months post-intervention.

Endpoints	Total, n (%)	Missing, n	Time	IG^†^, mean (SD)	CG^†^, mean (SD)	*p*-value between groups
Primary endpoint						
EQ-5D-3L (score)	53 (88.3)	7	3 months	2.4 (22.4)	1.4 (8.5)	0.841
	49 (81.7)	11	6 months	−2.7 (19.3)	−0.2 (9.2)	0.567
	49 (81.7)	11	9 months	−2.3 (15.1)	3.0 (11.7)	0.181
	47 (78.3)	13	10 months (follow-up)	6.9 (17.2)	5.9 (9.3)	0.814
VAS (score)	53 (88.3)	7	3 months	2.4 (23.2)	−4.9 (14.1)	0.191
	49 (81.7)	11	6 months	0.5 (22.4)	−5.0 (15.8)	0.332
	49 (81.7)	13	9 months	1.1 (22.0)	−4.7 (23.5)	0.386
	47 (78.3)	13	10 months (follow-up)	3.7 (21.2)	−1.6 (17.9)	0.369
Secondary endpoints						
Zarit Burden Inventory test (score)	53 (88.3)	7	3 months	−1.0 (5.4)	0.6 (5.2)	0.299
	49 (81.7)	11	6 months	−0.8 (7.4)	0.3 (5.7)	0.560
	50 (83.3)	10	9 months	−0.9 (8.0)	0.2 (5.0)	0.581
	48 (80.0)	12	10 months (follow-up)	0.3 (7.8)	−3.0 (7.7)	0.162
Satisfaction with Life Scale (score)	38 (63.3)	22	3 months	1.0 (2.5)	−0.6 (4.6)	0.220
	35 (58.3)	25	6 months	1.9 (3.2)	−0.7 (3.1)	**0.020**
	36 (60.0)	24	9 months	1.1 (3.2)	−0.5 (3.3)	0.156
	35 (58.3)	25	10 months (follow-up)	0.6 (3.8)	0.4 (3.1)	0.904
General Happiness Questionnaire (score)	53 (88.3)	7	3 months	−0.1 (0.9)	−0.2 (0.7)	0.700
	49 (81.7)	11	6 months	−0.2 (1.0)	−0.3 (0.8)	0.735
	49 (81.7)	11	9 months	−0.1 (0.8)	−0.2 (1.0)	0.678
	47 (78.3)	13	10 months (follow-up)	−0.1 (1.1)	−0.1 (0.6)	0.985
Occupational Balance Questionnaire-E (score)	53 (88.3)	7	3 months	8.1 (10.9)	−2.2 (11.9)	**0.002**
	49 (81.7)	11	6 months	6.5 (10.1)	−1.9 (12.5)	**0.013**
	49 (81.7)	11	9 months	6.0 (10.6)	−1.4 (10.9)	**0.022**
	47 (78.3)	13	10 months (follow-up)	7.1 (15.0)	0.8 (9.4)	0.109
Rosenberg self-esteem scale (score)	38 (63.3)	22	3 months	1.6 (5.0)	−0.6 (4.4)	0.156
	45 (75.0)	15	6 months	0.3 (4.8)	0.9 (4.1)	0.670
	43 (71.7)	17	9 months	−0.2 (5.6)	−0.5 (5.1)	0.857
	45 (75.0)	15	10 months (follow-up)	0.9 (3.9)	−1.0 (4.2)	0.138
Duke-UNC-11 Functional Social Support Questionnaire (score)	53 (88.3)	7	3 months	1.1 (5.1)	−2.8 (8.5)	**0.043**
	49 (81.7)	11	6 months	0.2 (6.5)	−0.6 (7.7)	0.711
	49 (81.7)	11	9 months	1.4 (8.4)	−2.7 (8.3)	0.090
	47 (78.3)	13	10 months (follow-up)	1.5 (7.7)	−0.2 (9.7)	0.490
Family Apgar scale (score)	53 (88.3)	7	3 months	−0.2 (1.5)	−0.2 (2.6)	0.975
	50 (83.3)	10	6 months	−0.6 (2.0)	−0.3 (2.8)	0.587
	49 (81.7)	11	9 months	0.4 (1.8)	0.0 (2.4)	0.583
	46 (76.7)	14	10 months (follow-up)	0.2 (1.6)	0.1 (2.4)	0.761

CG, control group; EQ-5D-3L, EuroQol-5 Dimensions-3 Levels; IG, intervention group; SD, standard deviation; VAS, visual analogue scale. Bold indicates a *p*-value < 0.05. ^†^Within-group difference from the baseline value.

The results obtained for each variable in each of the evaluations, as well as the difference between groups are also shown in Table 3. Significant differences were found at 3 (*p* = 0.002), 6 (*p* = 0.013) and 9 months (*p* = 0.022) for Occupational Balance Questionnaire-E, in perceived social support at 3 months (*p* = 0.043) and in life satisfaction at 6 months after the intervention (*p* = 0.020).

## 4 Discussion

The psycho-emotional, occupational and physical implications experienced by caregivers of people with AD have been widely documented in the scientific literature (Rodriguez-Mora et al., 2023; Nikzad-Terhune et al., 2019; Hernández-Padilla et al., 2021). However, this study adopted an original approach by not simply assessing these implications, but by implementing an intervention programme to compare its effectiveness in mitigating these adverse effects. This study is part of a larger project, and the objective data obtained from our results are essential to determine the financial feasibility and effectiveness of the intervention, which in turn would allow informed policy decisions in the field of health and prevention. To this end, we analysed the effect of the intervention over different timeframes based on participants’ baseline data to identify skills and resources to improve the emotional and psychological state of informal caregivers of people with AD, increasing quality of life and satisfaction.

In the scientific literature, studies were found that analysed the quality of life of caregivers of people with leukaemia (Yu et al., 2018) and cancer (Al-Rabayah et al., 2022), however, research of people with AD is scare. Despite this, one study analysed the quality of life of caregivers of people with dementia, showing very similar results to those found in our research and found no significant differences in caregiver’s quality of life, neither at 3 nor 6 months, showing a greater decrease in IG scores than CG (Birkenhäger-Gillesse et al., 2020). This could be because although caregivers have acquired knowledge and strategies for self-care and caregiving, they still must cope with the illness situation at home.

Regarding caregiver overload, our results did not identify any significant differences, although there is a clear trend towards a decrease in the values of this test over time, showing how caregiver overload is decreasing. One of the reasons why participants may have reduced their caregiver burden is the social development they have experienced, which is a source of resilience against symptoms of anxiety and depression, and they may experience less burden and higher quality of life (Amorim et al., 2017). Moreover, given the interdisciplinary nature of this intervention, having received training that enables family members to care for the sick with a greater degree of information, may also have led to a reduction in overload, as untrained caregivers with a high degree of misinformation can lead to overload (Cerquera Córdoba et al., 2012). Finally, it could also be attributed to certain inherent limitations of the instrument, such as the subjectivity of the responses, the lack of consideration of the context surrounding the caregiver, or the lack of discrimination between professional and non-professional caregivers.

The results in terms of happiness and life satisfaction, as well as self-perception or self-esteem, also showed no significant differences after the intervention. One possible explanation could be that, although the programme includes multiple tools aimed at improving caregivers’ self-care and quality of life, participation in these activities could lead to increased awareness of the seriousness of the situation, increased perception of burden and stress, as well as feelings of comparison with other more or less favourable realities (Cheng et al., 2019). Also, the relationship between patients and their relatives was not assessed during the study, as the relationship between them can be considered a relevant factor in determining life satisfaction in both (Rippon et al., 2020). These factors may limit the study’s ability to detect significant improvements.

Our baseline results, in terms of family functioning, suggest a moderate perception of dysfunction in the IG and CG, with homogeneity in both groups. However, in the evolution of the results with the intervention, a temporary improvement in the scores of the IG was observed. Increases in family functioning also have an impact on life satisfaction, according to another published study regarding the perception of family functioning and general life satisfaction. These results are consistent with those by Cabral et al., 2014, which show increases in IG, although they were not significant (Cabral et al., 2014).

In caregivers, knowledge, perceived social support and self-efficacy are among the protective variables for caregivers’ health (Sołtys and Tyburski, 2020; Tan et al., 2021). In our study, in the first 3 months of intervention the perceived social support improved in IG, however, this improvement was not significant in 6 and 9 months. This could be due to during the first 3 months new social relationships are established and companionship increasing, but over the months, the caregivers’ task is maintained or increased. A broad social network, information and social and emotional support for caregivers of older people with dementia is therefore required (Karg et al., 2018; Sandoval et al., 2019; Vullings et al., 2020). For this, sources of support and care plans related to the health of informal caregivers are necessary (De Maria et al., 1982). In this sense, one option to improve the carer’s perception of support would be the development of accessible, innovative and cost-effective methods of support. A new era of support could be developed through the internet and social networks, as physical limitations would be avoided, providing accessibility and independence in the home environment (Slegers and van Boxtel, 2013). Previous studies have shown the promising effects of online training and support programmes (Boots et al., 2014), in particular e-health interventions tailored to the needs of the caregiver, which could provide a positive experience and be more likely to be accepted by the caregiver (Boots et al., 2015).

Regarding occupations and occupational balance, significant differences were observed between the IG and CG at 3, 6 and 9 months, but not at subsequent follow-up. However, the intervention group shows an improvement in all four-study time. One study among caregivers highlighted the demands of caregiving and how it takes up all their time, resulting in caregiver exhaustion and forgetfulness. The approach used in this investigation was similar to that used in our research Through an intervention, they had the necessary strategies to prioritise activities and time for themselves (Watford et al., 2019). Thus, the results were similar to those reported in our study since the IG can adjust and even improve the OBQ score over the months, compared to the CG. These results suggest an improvement in their perception of occupations over time and support the idea of the duration of the intervention to achieve positive results, even at the 10-month follow-up Nevertheless, another study linked occupational balance impairment and physical disease obtained non-significant results when assessing pharmacological intervention, understanding that occupational balance is a subjective process that depends on the individual and is related to the number and variety of occupations. Previous studies addressing occupational balance after deprivation due to various causes, such as illness or confinement, reflected the difficulty of restoring occupational activities. They also showed the importance of focusing on occupational balance (Rodríguez-Rivas et al., 2022; Kassberg et al., 2021). Previous studies have pointed out the need to help monitor activity patterns and perceived balance to subsequently adopt activity-based strategies to promote quality of life and subjective health perception, which may even have an impact on the quality of care provided (Röschel et al., 2022; Davy et al., 2024; Davy et al., 2022). Our results are in line with the idea that occupational balance is a dynamic process in which caregivers’ adaptive capacities help to restore occupational balance (Mahdizadeh et al., 2023).

It is noteworthy that this study follows the pattern of femininity in care expressed in previous studies (Liu et al., 2024; Cerquera Córdoba et al., 2021; Peña-Longobardo and Oliva-Moreno, 2015). In fact, more than three quarters of the population studied is female.

Among the main limitations of this study were, firstly, the size of the sample (*n* = 59), which could affect the statistical power of the results and ultimately the statistical significance obtained. Secondly, the lack of assessment of the caregiver context and possible caregiver-patient relationships. Additionally, the collection of further caregiving support, including from family and friends, could have helped to understand the results of our study. Finally, the use of self-reported questionnaires could introduce social desirability and recall biases.

However, we found great strengths in this research. Objective and quantifiable data were collected on an innovative programme designed to ensure that caregivers manage the situation and find a balance between caregiving and their health and happiness. In addition, the repeated evaluation of different measures, as well as the follow-up, allowed us to verify and contrast in a more accurate way the results obtained during the different evaluations carried out temporarily and at the end of the intervention and thus the evaluation process.

## 5 Conclusion

A nine-month interdisciplinary programme based on cognitive-behavioural, psychoemotional, and physical activity can significantly increase occupational balance in informal caregivers of people with AD. No significant improvements were found in the quality of life, physical, emotional, and social overload and family functioning, although there were positive trends in the intra-group scores for caregivers overload, quality of life, life satisfaction and family functioning.

This study represents the first step in objectively assessing the cost-effectiveness of such programmes. However, further research is needed to study more variables and to measure over longer periods to identify grasps and observe long-term benefits.

## Data Availability

The original contributions presented in the study are included in the article/supplementary material, further inquiries can be directed to the corresponding authors.

## Footnotes

^1^ https://www.integral-care.es/login

## References

[ref1] Allen A. P. Buckley M. M. Cryan J. F. Ni Chorcorain A. Dinan T. G. Kearney P. M.. (2019). Informal caregiving for dementia patients: the contribution of patient characteristics and behaviours to caregiver burden. Age Ageing 49, 52–56. doi: 10.1093/ageing/afz128, PMID: 31755532

[ref2] Al-Rabayah A. A. Al Froukh R. F. Al Najjar B. Rayyan M. Salmany S. Iweir S.. (2022). Quality of life of family caregivers of critically ill patients with Cancer before and after intensive care unit admission measured by EQ-5D 3-level: a longitudinal prospective cohort study. Value Health Reg Issues. 30, 39–47. doi: 10.1016/j.vhri.2021.11.003, PMID: 35086001

[ref3] Amorim F. A. Giorgion M. C. P. Forlenza O. V. (2017). Social skills and well-being among family caregivers to patients with Alzheimer’s disease. Archives Clin. Psychiatry (São Paulo). 44, 159–161. doi: 10.1590/0101-60830000000143

[ref4] An H. Y. Chen W. Wang C. W. Yang H. F. Huang W. T. Fan S. Y. (2020). The relationships between physical activity and life satisfaction and happiness among young, middle-aged, and older adults. Int. J. Environ. Res. Public Health 17:4817. doi: 10.3390/ijerph17134817, PMID: 32635457 PMC7369812

[ref5] Association As. (2024). Alzheimer's disease facts and figures. Alzheimers Dement. 20, 3708–3821. doi: 10.1002/alz.13809 38689398 PMC11095490

[ref6] Atri A. (2019). The Alzheimer's disease clinical Spectrum: diagnosis and management. Med. Clin. North Am. 103, 263–293. doi: 10.1016/j.mcna.2018.10.009, PMID: 30704681

[ref7] Bhattacharjee M. Vairale J. Gawali K. Dalal P. M. (2012). Factors affecting burden on caregivers of stroke survivors: population-based study in Mumbai (India). Ann. Indian Acad. Neurol. 15, 113–119. doi: 10.4103/0972-2327.94994, PMID: 22566724 PMC3345587

[ref8] Birkenhäger-Gillesse E. G. Achterberg W. P. Janus S. I. Kollen B. J. (2020). Effects of caregiver dementia training in caregiver-patient dyads: A randomized controlled study. Int. J. Geriatric Psychiatry. 35, 1376–1384. doi: 10.1002/gps.5378, PMID: 32662184 PMC7689696

[ref9] Boots L. M. de Vugt M. E. van Knippenberg R. J. Kempen G. I. Verhey F. R. (2014). A systematic review of internet-based supportive interventions for caregivers of patients with dementia. Int. J. Geriatr. Psychiatry 29, 331–344. doi: 10.1002/gps.4016, PMID: 23963684

[ref10] Boots L. M. Wolfs C. A. Verhey F. R. Kempen G. I. de Vugt M. E. (2015). Qualitative study on needs and wishes of early-stage dementia caregivers: the paradox between needing and accepting help. Int. Psychogeriatr. 27, 927–936. doi: 10.1017/S1041610214002804, PMID: 25566686

[ref11] Broadhead W. E. Gehlbach S. H. de Gruy F. V. Kaplan B. H. (1988). The Duke-UNC functional social support questionnaire. Measurement of social support in family medicine patients. Med. Care 26, 709–723. doi: 10.1097/00005650-198807000-00006, PMID: 3393031

[ref12] Brodaty H. Donkin M. (2009). Family caregivers of people with dementia. Dialogues Clin. Neurosci. 11, 217–228. doi: 10.31887/DCNS.2009.11.2/hbrodaty, PMID: 19585957 PMC3181916

[ref13] Cabral L. Duarte J. Ferreira M. dos Santos C. (2014). Ansiedad, estrés y depresión en cuidadores familiares de enfermos mentales. Aten Primaria. (Suppl 5) 46, 176–179. doi: 10.1016/S0212-6567(14)70087-3, PMID: 25476057 PMC8171474

[ref14] Cerquera Córdoba A. M. Granados Latorre F. J. Buitrago Mariño A. M. (2012). Overload in caregivers for patients with Alzheimer dementia. Psychologia Avances de la disciplina. 6, 35–45.

[ref15] Cerquera Córdoba A. M. Tiga Loza D. C. Álvarez Anaya W. A. Dugarte Peña E. Jaimes Espíndola L. R. (2021). Ensayo controlado aleatorizado de un programa multicomponente para cuidadores informales de pacientes con Alzheimer. Revista Cuidarte 12:e2002. doi: 10.15649/cuidarte.2002

[ref16] Cheng S. T. (2017). Dementia caregiver burden: a research update and critical analysis. Curr. Psychiatry Rep. 19:64. doi: 10.1007/s11920-017-0818-2, PMID: 28795386 PMC5550537

[ref17] Cheng S. T. Au A. Losada A. Thompson L. W. Gallagher-Thompson D. (2019). Psychological interventions for dementia caregivers: what we have achieved, what we have learned. Curr. Psychiatry Rep. 21:59. doi: 10.1007/s11920-019-1045-9, PMID: 31172302 PMC6554248

[ref18] Cho D. (2017). Analytical concept of happiness and its measurement. J. Labour Econ. 40, 79–104.

[ref19] Collaborators GBDDF (2022). Estimation of the global prevalence of dementia in 2019 and forecasted prevalence in 2050: an analysis for the global burden of disease study 2019. Lancet Public Health 7, e105–e125. doi: 10.1016/S2468-2667(21)00249-8 34998485 PMC8810394

[ref20] Cui M. Y. Lin Y. Sheng J. Y. Zhang X. Cui R. J. (2018). Exercise intervention associated with cognitive improvement in Alzheimer's disease. Neural Plast. 2018, 1–10. doi: 10.1155/2018/9234105, PMID: 29713339 PMC5866875

[ref21] Davy G. Barbaro J. Unwin K. Clark M. Jellett R. Date P.. (2024). Leisure, community, workforce participation and quality of life in primary and secondary caregivers of autistic children. Autism Res. 17, 799–811. doi: 10.1002/aur.3113, PMID: 38414177

[ref22] Davy G. Unwin K. L. Barbaro J. Dissanayake C. (2022). Leisure, employemnt, community participation and quality of life in caregivers of austic children: a scoping review. Autism 26, 1916–1930. doi: 10.1177/13623613221105836, PMID: 35765798

[ref23] De la Rosa A. Olaso-Gonzalez G. Arc-Chagnaud C. Millan F. Salvador-Pascual A. Garcia-Lucerga C.. (2020). Physical exercise in the prevention and treatment of Alzheimer's disease. J. Sport Health Sci. 9, 394–404. doi: 10.1016/j.jshs.2020.01.004, PMID: 32780691 PMC7498620

[ref24] De Maria M. Tagliabue S. Ausili D. Vellone E. Matarese M. (1982). Perceived social support and health-related quality of life in older adults who have multiple chronic conditions and their caregivers: a dyadic analysis. Soc. Sci. Med. 262:113193. doi: 10.1016/j.socscimed.2020.113193, PMID: 32777671

[ref25] de Rotrou J. Cantegreil I. Faucounau V. Wenisch E. Chausson C. Jegou D.. (2011). Do patients diagnosed with Alzheimer's disease benefit from a psycho-educational programme for family caregivers? A randomised controlled study. Int. J. Geriatr. Psychiatry 26, 833–842. doi: 10.1002/gps.2611, PMID: 20922772

[ref26] Diener E. Emmons R. A. Larsen R. J. Griffin S. (1985). The satisfaction with life scale. J. Pers. Assess. 49, 71–75. doi: 10.1207/s15327752jpa4901_13, PMID: 16367493

[ref27] Evans K. A. (1987). Definition of occupation as the core concept of occupational therapy. Am. J. Occup. Ther. 41, 627–628. doi: 10.5014/ajot.41.10.627, PMID: 3688116

[ref28] Freedman V. A. Spillman B. C. (2014). Disability and care needs among older americans. Milbank Q. 92, 509–541. doi: 10.1111/1468-0009.12076, PMID: 25199898 PMC4221755

[ref29] Gómez P. P. (2017). Equilibrio ocupacional en Estudiantes de Terapia Ocupacional. Elche, Spain: Universidad Miguel Hernández.

[ref30] Gomez Gallego M. Gomez G. J. (2017). Music therapy and Alzheimer's disease: cognitive, psychological, and behavioural effects. Neurologia 32, 300–308. doi: 10.1016/j.nrl.2015.12.003, PMID: 26896913

[ref31] Herdman M. Gudex C. Lloyd A. Janssen M. Kind P. Parkin D.. (2011). Development and preliminary testing of the new five-level version of EQ-5D (EQ-5D-5L). Qual. Life Res. 20, 1727–1736. doi: 10.1007/s11136-011-9903-x, PMID: 21479777 PMC3220807

[ref32] Hernández-Gúzman L. Dobson K. S. Caso-Niebla J. González-Montesinos M. Epp A. Arratíbel-Siles M. L.. (2009). La versión en Español de la escala cognitivo-conductual de evitación (CBAS). Rev Latinoam Psicol. 41, 99–108.

[ref33] Hernández-Padilla J. M. Ruiz-Fernández M. D. Granero-Molina J. Ortíz-Amo R. López Rodríguez M. M. Fernández-Sola C. (2021). Perceived health, caregiver overload and perceived social support in family caregivers of patients with Alzheimer's: gender differences. Health Soc. Care Community 29, 1001–1009. doi: 10.1111/hsc.13134, PMID: 32783241

[ref34] Karg N. Graessel E. Randzio O. Pendergrass A. (2018). Dementia as a predictor of care-related quality of life in informal caregivers: a cross-sectional study to investigate differences in health-related outcomes between dementia and non-dementia caregivers. BMC Geriatr. 18:189. doi: 10.1186/s12877-018-0885-1, PMID: 30139354 PMC6108112

[ref35] Kasper J. D. Freedman V. A. Spillman B. C. Wolff J. L. (2015). The disproportionate impact of dementia on family and unpaid caregiving to older adults. Health Aff (Millwood). 34, 1642–1649. doi: 10.1377/hlthaff.2015.0536 26438739 PMC4635557

[ref36] Kassberg A. C. Nyman A. Larsson L. M. (2021). Perceived occupational balance in people with stroke. Disabil. Rehabil. 43, 553–558. doi: 10.1080/09638288.2019.1632940, PMID: 31264487

[ref37] Kim D. (2020). The effects of a recollection-based occupational therapy program of Alzheimer's disease: a randomized controlled trial. Occup. Ther. Int. 2020, 1–8. doi: 10.1155/2020/6305727, PMID: 32821251 PMC7416254

[ref38] Kim J. Lee J. Ko M. J. Min O. S. (2022). Leisure, mental health, and life satisfaction among older adults with mild cognitive impairment. Am. J. Health Behav. 46, 477–487. doi: 10.5993/AJHB.46.4.8, PMID: 36109858

[ref39] Lee C. D. Kim D. Lee M. J. Kang J. Foster E. R. (2023). The relationship between active, balanced participation and well-being in older adults in the United States: a time-use perspective. J. Occup. Sci. 30, 175–183. doi: 10.1080/14427591.2020.1869584, PMID: 39989647

[ref40] Leggett A. N. Meyer O. L. Bugajski B. C. Polenick C. A. (2021). Accentuate the positive: the association between informal and formal supports and caregiving gains. J. Appl. Gerontol. 40, 763–771. doi: 10.1177/0733464820914481, PMID: 32326797 PMC7584731

[ref41] Lin J. D. Lin P. Y. Wu C. L. (2010). Wellbeing perception of institutional caregivers working for people with disabilities: use of subjective happiness scale and satisfaction with life scale analyses. Res. Dev. Disabil. 31, 1083–1090. doi: 10.1016/j.ridd.2010.03.009, PMID: 20400262

[ref42] Liu X. Wang S. Wei L. Liu Y. Bian J. Wang S.. (2024). The impact of empowerment theory-based health education on Alzheimer's disease informal caregivers: a randomized controlled trial. Front. Public Health 12:1393823. doi: 10.3389/fpubh.2024.1393823, PMID: 39257940 PMC11385866

[ref43] Lyubomirsky S. Sheldon K. M. Schkade D. (2005). Pursuing happiness: the architecture of sustainable change. Rev. Gen. Psychol. 9, 111–131. doi: 10.1037/1089-2680.9.2.111

[ref44] Lyumbornirsky S. Lepper H. S. (1999). A measure of subjective happiness: preliminary reliability and construct validation. Soc. Indic. Res. 46, 137–155. doi: 10.1023/A:1006824100041, PMID: 38124636

[ref45] Mahdizadeh A. Khankeh H. Ghodsi H. Hosseini S. A. Akbarfahimi N. (2023). Post-COVID-19 survivors’ strategies for improving occupational balance: a qualitative study. Br. J. Occup. Ther. 86, 777–786. doi: 10.1177/03080226231184708

[ref46] Martens L. Addington J. (2001). The psychological well-being of family members of individuals with schizophrenia. Soc. Psychiatry Psychiatr. Epidemiol. 36, 128–133. doi: 10.1007/s001270050301, PMID: 11465784

[ref47] Meziane-Damnée S. Bayle C. Pino M. Lenoir H. Cantegreil L. Rigaud A. S. (2023). Un programme psychoéducatif pour les aidants familiaux de personnes souffrant d'Alzheimer entrant en institution. Sains Gérontologie. 28, 20–23. doi: 10.1016/j.sger.2023.04.007, PMID: 37328202

[ref48] Milovanska-Farrington S. Farrington S. (2022). Happiness, domains of life satisfaction, perceptions, and valuation differences across genders. Acta Psychol. 230:103720. doi: 10.1016/j.actpsy.2022.103720 36084437

[ref49] Morejón A. J. García-Bóveda R. J. Jímenez R. V. M. (2004). Escala de autoestima de Rosenberg: Fiabilidad y validez en población clínica española. Apunt Psicol. 22, 247–255. doi: 10.55414/bsxyn321

[ref50] Morhardt D. J. O'Hara M. C. Zachrich K. Wieneke C. Rogalski E. J. (2019). Development of a psycho-educational support program for individuals with primary progressive aphasia and their care-partners. Dementia (London). 18, 1310–1327. doi: 10.1177/1471301217699675, PMID: 29149795 PMC5696112

[ref51] Muñoz-Bermejo L. González-Becerra M. J. Barrios-Fernandez S. Postigo-Mota S. Jerez-Barroso M. R. Martínez J. A. E.. (2022). Cost-effectiveness of the comprehensive interdisciplinary program-Care in Informal Caregivers of people with Alzheimer's disease. Int. J. Environ. Res. Public Health 19:15243. doi: 10.3390/ijerph192215243, PMID: 36429962 PMC9691117

[ref52] Nemcikova M. Katreniakova Z. Nagyova I. (2023). Social support, positive caregiving experience, and caregiver burden in informal caregivers of older adults with dementia. Front. Public Health 11:1104250. doi: 10.3389/fpubh.2023.1104250, PMID: 36761127 PMC9905841

[ref53] Nikzad-Terhune K. Gaugler J. E. Jacobs-Lawson J. (2019). Dementia caregiving outcomes: the impact of caregiving onset, cognitive impairment and behavioral problems. J. Gerontol. Soc. Work. 62, 543–563. doi: 10.1080/01634372.2019.1625993, PMID: 31166157

[ref54] Ong H. L. Vaingankar J. A. Abdin E. Sambasivam R. Fauziana R. Tan M. E.. (2018). Resilience and burden in caregivers of older adults: moderating and mediating effects of perceived social support. BMC Psychiatry 18:27. doi: 10.1186/s12888-018-1616-z, PMID: 29385985 PMC5793423

[ref55] Park S. Lee H. J. Jeon B. J. Yoo E. Y. Kim J. B. Park J. H. (2021). Effects of occupational balance on subjective health, quality of life, and health-related variables in community-dwelling older adults: a structural equation modeling approach. PLoS One 16:e0246887. doi: 10.1371/journal.pone.0246887, PMID: 33571290 PMC7877622

[ref56] Peña-Longobardo L. M. Oliva-Moreno J. (2015). Caregiver burden in Alzheimer's disease patients in Spain. J. Alzheimer's Dis: JAD. 43, 1293–1302. doi: 10.3233/JAD-141374, PMID: 25159671

[ref57] Rabarison K. M. Bouldin E. D. Bish C. L. McGuire L. C. Taylor C. A. Greenlund K. J. (2018). The economic value of informal caregiving for persons with dementia: results from 38 states, the District of Columbia, and Puerto Rico, 2015 and 2016 BRFSS. Am. J. Public Health 108, 1370–1377. doi: 10.2105/AJPH.2018.304573, PMID: 30138069 PMC6137771

[ref58] Rippon I. Quinn C. Martyr A. Morris R. Nelis S. M. Jones I. R.. (2020). The impact of relationship quality on life satisfaction and well-being in dementia caregiving dyads: findings from the IDEAL study. Aging Ment. Health 24, 1411–1420. doi: 10.1080/13607863.2019.1617238, PMID: 31140291

[ref59] Rodriguez-Mora A. Mateo Guirola T. Mestre J. M. (2023). Overload and emotional wellbeing in a sample of Spanish caregivers of Alzheimer's patients during COVID-19 pandemic. Exp. Aging Res. 49, 389–406. doi: 10.1080/0361073X.2022.2115739, PMID: 36036728

[ref60] Rodríguez-Rivas C. Camacho-Montaño L. R. García-Bravo C. García-de-Miguel M. Pérez-de-Heredia-Torres M. Huertas-Hoyas E. (2022). Effects of social isolation measures caused by the COVID-19 pandemic on occupational balnce, participation and activities' satisfaction in the Spanish population. Int. J. Environ. Res. Public Health 19:6497. doi: 10.3390/ijerph19116497, PMID: 35682080 PMC9180883

[ref61] Röschel A. Wagner C. Dür M. (2022). Associations between occupational balance, subjective health, and well-being of informal caregivers of older persons based on a cross-sectional study. BMC Geriatr. 22:445. doi: 10.1186/s12877-022-03124-1, PMID: 35596125 PMC9123703

[ref62] Sallim A. B. Sayampanathan A. A. Cuttilan A. Ho R. (2015). Prevalence of mental health disorders among caregivers of patients with Alzheimer disease. J. Am. Med. Dir. Assoc. 16, 1034–1041. doi: 10.1016/j.jamda.2015.09.007, PMID: 26593303

[ref63] Sandoval F. Tamiya N. Lloyd-Sherlock P. Noguchi H. (2019). The relationship between perceived social support and depressive symptoms in informal caregivers of community-dwelling older persons in Chile. Psychogeriatrics: the official journal of the Japanese psychogeriatric. Society 19, 547–556. doi: 10.1111/psyg.12438, PMID: 30864201 PMC6900012

[ref64] Slegers K. van Boxtel M. P. (2013). “Actual use of computers and the internet by older adults: potential benefits and risks” in Engaging older adults with modern technology: Internet use and information access needs. eds. Zheng R. Z. Hill R. D. Gardner M. K. (US: IGI Global), 161–190.

[ref65] Sołtys A. Tyburski E. (2020). Predictors of mental health problems in formal and informal caregivers of patients with Alzheimer's disease. BMC Psychiatry 20:435. doi: 10.1186/s12888-020-02822-7, PMID: 32887576 PMC7487573

[ref66] Tan G. T. H. Yuan Q. Devi F. Wang P. Ng L. L. Goveas R.. (2021). Factors associated with caregiving self-efficacy among primary informal caregivers of persons with dementia in Singapore. BMC Geriatr. 21:13. doi: 10.1186/s12877-020-01951-8, PMID: 33407201 PMC7789728

[ref67] Thunyadee C. Sitthimongkol Y. Sangon S. Chai-Aroon T. Hegadoren K. M. (2015). Predictors of depressive symptoms and physical health in caregivers of individuals with schizophrenia. Nurs. Health Sci. 17, 412–419. doi: 10.1111/nhs.12205, PMID: 26081195

[ref68] Vullings I. Labrie N. Wammes J. D. de Bekker-Grob E. W. Mac N.-V. J. (2020). Important components for Dutch in-home care based on qualitative interviews with persons with dementia and informal caregivers. Health Expectations: Int. J. Public Participation Health Care Health Policy. 23, 1412–1419. doi: 10.1111/hex.13118, PMID: 33026139 PMC7752200

[ref69] Watford P. Jewell V. Atler K. (2019). Increasing meaningful occupation for women who provide Care for Their Spouse: a pilot study. OTJR (Thorofare NJ). 39, 213–221. doi: 10.1177/1539449219829849, PMID: 30810080

[ref70] WHO (2000). Obesity: preventing and managing the global epidemic. Report of a WHO Consulation. 894, 1–253. 11234459

[ref71] Wiegelmann H. Speller S. Verhaert L. M. Schirra-Weirich L. Wolf-Ostermann K. (2021). Psychosocial interventions to support the mental health of informal caregivers of persons living with dementia - a systematic literature review. BMC Geriatr. 21:94. doi: 10.1186/s12877-021-02020-4, PMID: 33526012 PMC7849618

[ref72] Williams J. B. Cao Q. Yan Z. (2021). Transcriptomic analysis of human brains with Alzheimer's disease reveals the altered expression of synaptic genes linked to cognitive deficits. Brain Commun. 3:fcab123. doi: 10.1093/braincomms/fcab123, PMID: 34423299 PMC8374979

[ref73] Yu H. Zhang H. Yang J. Liu C. Lu C. Yang H.. (2018). Health utility scores of family caregivers for leukemia patients measured by EQ-5D-3L: a cross-sectional survey in China. BMC Cancer 18:950. doi: 10.1186/s12885-018-4855-y, PMID: 30285666 PMC6171222

[ref74] Zarit S. H. Reever K. E. Bach-Peterson J. (1980). Relatives of the impaired elderly: correlates of feelings of burden. Gerontology 20, 649–655. 10.1093/geront/20.6.649 7203086

